# The celecoxib derivatives AR-12 and AR-14 induce autophagy and clear prion-infected cells from prions

**DOI:** 10.1038/s41598-017-17770-8

**Published:** 2017-12-14

**Authors:** Basant A. Abdulrahman, Dalia Abdelaziz, Simrika Thapa, Li Lu, Shubha Jain, Sabine Gilch, Stefan Proniuk, Alexander Zukiwski, Hermann M. Schatzl

**Affiliations:** 1 Department of Comparative Biology & Experimental Medicine, Faculty of Veterinary Medicine, University of Calgary, Calgary, Alberta T2N 4Z6 Canada; 2 Department of Ecosystem & Public Health, Faculty of Veterinary Medicine, University of Calgary, Calgary, Alberta T2N 4Z6 Canada; 3 Calgary Prion Research Unit, University of Calgary, Calgary, Alberta T2N 4Z6 Canada; 4 Department of Biochemistry & Molecular Biology, Faculty of Pharmacy, Helwan University, 11795 Cairo, Egypt; 5 Departments of Veterinary Sciences and of Molecular Biology, University of Wyoming, Laramie, Wyoming, 82071 USA; 6 Arno Therapeutics, Inc., Flemington, NJ USA

## Abstract

Prion diseases are fatal infectious neurodegenerative disorders that affect both humans and animals. The autocatalytic conversion of the cellular prion protein (PrP^C^) into the pathologic isoform PrP^Sc^ is a key feature in prion pathogenesis. AR-12 is an IND-approved derivative of celecoxib that demonstrated preclinical activity against several microbial diseases. Recently, AR-12 has been shown to facilitate clearance of misfolded proteins. The latter proposes AR-12 to be a potential therapeutic agent for neurodegenerative disorders. In this study, we investigated the role of AR-12 and its derivatives in controlling prion infection. We tested AR-12 in prion infected neuronal and non-neuronal cell lines. Immunoblotting and confocal microscopy results showed that AR-12 and its analogue AR-14 reduced PrP^Sc^ levels after only 72 hours of treatment. Furthermore, infected cells were cured of PrP^Sc^ after exposure of AR-12 or AR-14 for only two weeks. We partially attribute the influence of the AR compounds on prion propagation to autophagy stimulation, in line with our previous findings that drug-induced stimulation of autophagy has anti-prion effects *in vitro* and *in vivo*. Taken together, this study demonstrates that AR-12 and the AR-14 analogue are potential new therapeutic agents for prion diseases and possibly protein misfolding disorders involving prion-like mechanisms.

## Introduction

The highly conserved *PRNP/Prnp* gene encodes the cellular prion protein (PrP^C^), a protein highly expressed in the central nervous system in neurons and glial cells, and present in non-brain cells. The exact physiological role of PrP^C^ is a matter of debate^1–4^. In prion diseases, PrP^C^ is converted into the pathological isoform PrP^Sc^ that is infectious in the absence of encoding nucleic acid^5,6^. Subsequent accumulation of PrP^Sc^ leads to a series of fatal neurodegenerative diseases in humans and animals. Human prion diseases include the various forms of Creutzfeldt-Jakob disease (CJD), Gerstmann-Sträussler-Scheinker syndrome (GSS), and fatal familial insomnia (FFI). Animal prion diseases are scrapie in sheep and goats, bovine spongiform encephalopathy (BSE) in cattle and other species, and chronic wasting disease (CWD) in cervids^7–10^. Loss of neurons, astrogliosis and mild microglia activation are the main pathological features of prion diseases. This results in a progressive spongiform degeneration of the central nervous system (CNS), leading to ataxia, behavioral changes and, in humans, highly progressive loss of intellectual abilities^6,11–13^. In the last two decades, great efforts have been made to establish treatment options for prion diseases. These included testing existing drugs for anti-prion activity in experimental models^14–21^ with only a few agents progressing to human studies of patients with prion diseases^22–25^. Investigations to date have not resulted in a recognized/proven treatment for prion diseases.

AR-12 (a.k.a. OSU-03012) is an antitumor celecoxib-derivative that lacks cyclooxygenase-2 (COX-2) inhibitor activity. It inhibits phosphoinositide-dependent kinase-1 (PDK1) activity in different cell models and a first human clinical trial has been completed^26–30^. Interestingly, it shows activity against a number of infectious agents including bacteria, fungi and viruses^31–35^. It is an orally available small molecule with human safety data and is known to cross effectively the blood-brain barrier^36^. Mechanistic studies suggest that AR-12 down-regulates the host cell chaperone machinery, preventing proper folding of viral proteins and efficient viral assembly^37^. Additionally, AR-12 has been shown to down-regulate GRP78, resulting in up-regulation of Atg13 and PERK, which induces autophagy and facilitates the clearance of intracellular viruses and/or unfolded proteins^38^. We have reported that drug-induced autophagy stimulation has anti-prion effects *in vitro* and *in vivo* ^20,21,39^. In our search for compounds which induce autophagy and reduce prions, we tested AR-12 and several analogues in two prion-infected neuronal cell lines (N2a and CAD5) and prion-infected mouse embryonic fibroblasts (MEFs). AR-12 significantly decreased PrP^Sc^ levels in all three prion-infected cell lines after three days of treatment with a single dose of the compound. Our findings were confirmed by PrP^Sc^-specific immunofluorescence analysis and real-time quaking-induced conversion (RT-QuIC), where application of AR-12 and its analogue AR-14 to infected cells resulted in a robust decrease in prion conversion activity. This very efficient anti-prion effect upon short-term treatment suggests that the compound may have multiple effects on prion propagation and/or acts on PrP^Sc^ clearance. In line with this, PrP^Sc^ decrease was accompanied by up-regulation of autophagy markers like LC3-II. Using prion-infected N2a cells with CRISPR/Cas9-based knock-out of *ATG5* gene, resulting in a loss of autophagy function, proved that autophagy is involved in the mode of anti-prion action of AR-12 and AR-14. Importantly, prolonged treatment with AR-12 and AR-14 for two weeks substantially cleared prion infection from ScN2a and ScMEF cells.

To our knowledge, this is the first report to investigate the role of AR-12 and AR-14 in prion-infected cells. Our data show that AR-12 and its derivatives could be promising therapeutic tools for the treatment of prion diseases and protein misfolding diseases.

## Results

### AR-12 controls prion infection in various prion cell culture models

**Figure 1:**
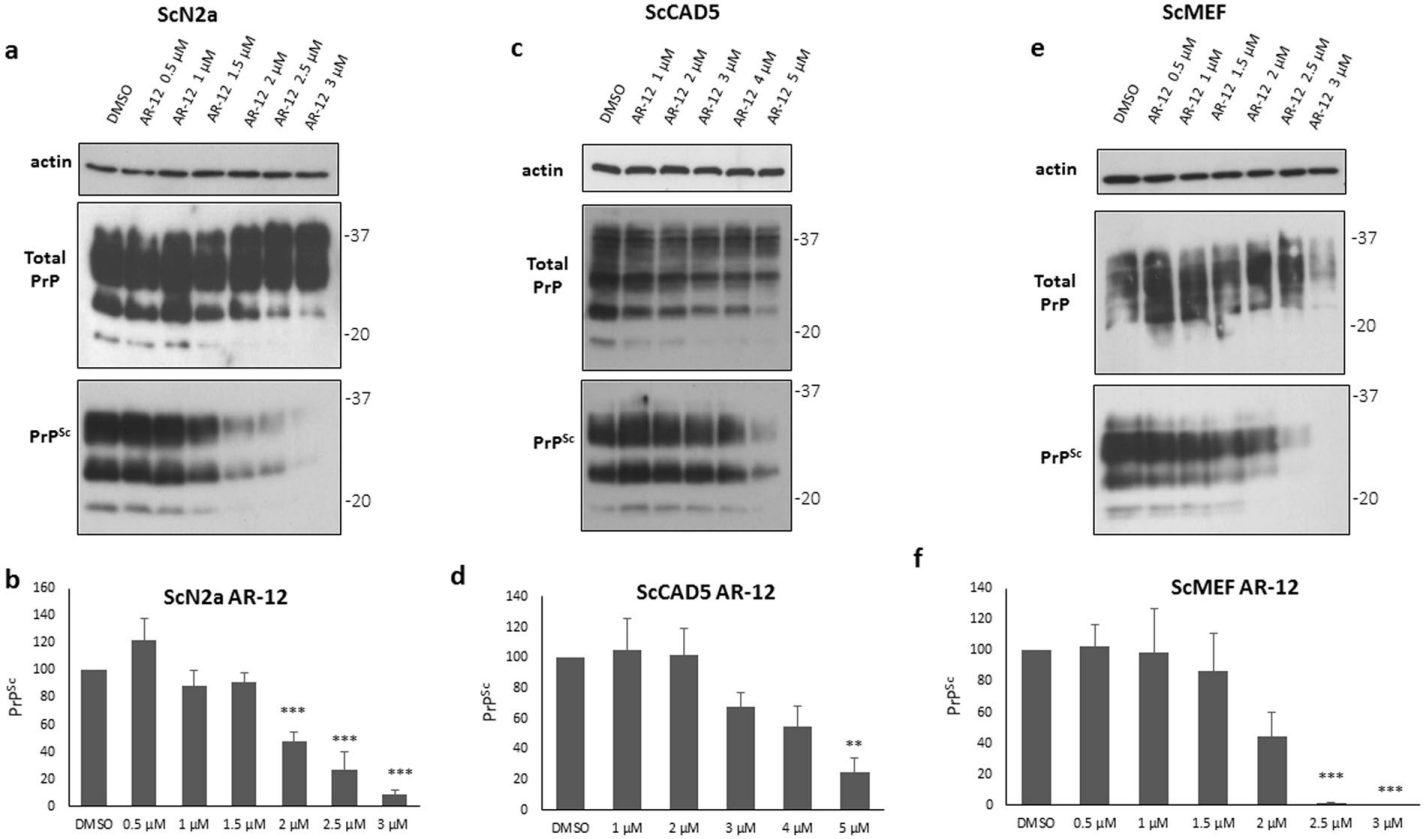
Short-term treatment with AR-12 reduces PrP^Sc^ levels in three persistently prion-infected cell lines. **(a)** Persistently prion-infected neuroblastoma cells (ScN2a; prion strain 22 L) were treated for 72 h with 0.5, 1, 1.5, 2, 2.5 or 3 µM of AR-12. Solvent only-treated cells (DMSO) were used as control. Cells were lysed and lysates split into two halves. One was treated with proteinase K (PK; 20 µg/ml for 30 min at 37 °C) and both subjected to immunoblot analysis. Immunoblot was developed with anti-PrP monoclonal antibody (mAb) 4H11 and the blot was re-probed with a mAb for actin (gel loading control). **(b)** Densitometric analysis of ScN2a immunoblots. Data are represented as a percentage of vehicle-treated (DMSO) cells from at least three independent experiments. **(c)** Persistently prion-infected neuronal ScCAD5 cells (prion strain 22 L) were treated for 72 h with 1, 2, 3, 4 or 5 µM of AR-12. Solvent only-treated cells (DMSO) were used as control. Immunoblot was developed with anti-PrP mAb 4H11 and the blot was re-probed for actin (gel loading). PrP^Sc^ was reduced after AR-12 treatment. **(d)** Densitometric analysis for ScCAD5 immunoblots. Data are represented as a percentage of vehicle-treated (DMSO) cells. **(e)** Persistently prion-infected mouse embryonic fibroblasts (MEF) (prion strain 22 L) were treated for 72 h with 0.5, 1,1.5, 2, 2.5 or 3 µM of AR-12 and analyzed as above. **(f)** Densitometric analysis for ScMEF immunoblots. Data are represented as a percentage of vehicle-treated (DMSO) cells. (*p < *0*.*05*), (**p < *0*.*01*) (***p < *0*.*001*) considered significant.

To address the effect of AR-12 in prion infected cells, we used three different cell lines. The murine neuroblastoma cell line ScN2a (infected with prion strain 22 L) of peripheral nervous system (PNS) origin^40^, the murine catecholaminergic/neuronal cell line ScCAD5 (infected with prion strain 22 L) of CNS origin^41^, and prion infected immortalized mouse embryonic fibroblasts ScMEF (22 L infected) as non-neuronal cells. In order to analyze whether AR-12 is affecting the level of PrP^Sc^ in ScN2a cells, we treated cells for 72 h with increasing concentrations of AR-12, from 0.5 to 3 µM, in a single application. A dose-dependent reduction of PrP^Sc^ was observed upon treatment. The effective dose 50% (EC_50_) was 1.5 µM **(**Fig. 1a,b **)**. Concentrations of 2, 2.5 and 3 µM of AR-12 significantly reduced PrP^Sc^ levels (p < 0.001). Of note, toxic effects were not observed when cells were treated with AR-12 under these conditions. Median lethal dose 50% (LD_50_) was 5 µM **(**Fig. S1a **)**. Next, we investigated the effect of AR-12 in the catecholaminergic/neuronal cell line ScCAD5, using a range of concentrations from 1 to 5 µM for 72 h. A concentration of 5 µM significantly enhanced the clearance of PrP^Sc^ (p < 0.01), with an EC_50_ of 4 µM **(**Fig. 1c,d **)**. Yet, the effect seemed less pronounced compared to the one in ScN2a cells. To exclude that the observed decrease in PrP^Sc^ was related to drug toxicity, cytotoxicity assays were conducted. The results showed a safety margin up to 5 µM of AR-12 for 72 h of treatment in ScCAD5 cells. LD_50_ was 9 µM **(**Fig. S1b **)**. Next, we tested whether the effect of AR-12 on PrP^Sc^ is limited to neuronal cell lines. We treated ScMEFs cells (infected with prion strain 22 L) with AR-12 at a range of concentrations from 0.5 to 3 µM for 72 h. A substantial decrease in PrP^Sc^ was observed at concentrations of 2.5 and 3 µM (p < 0.001). EC_50_ was 2 µM **(**Fig. 1e,f **)**. AR-12 treatment was non-toxic to ScMEF cells up to a concentration of 4 µM, with a LD50 of 5 µM **(**Fig. S1c **)**. Similar results were obtained with ScMEFs infected with strains prions RML and Me7 (data not shown).

Taken together, our data show that treatment with AR-12 enhances degradation of PrP^Sc^ in neuronal (ScN2a/ScCAD5) and non-neuronal cells (ScMEF).

### AR-14 promotes clearance of PrP^Sc^

**Figure 2:**
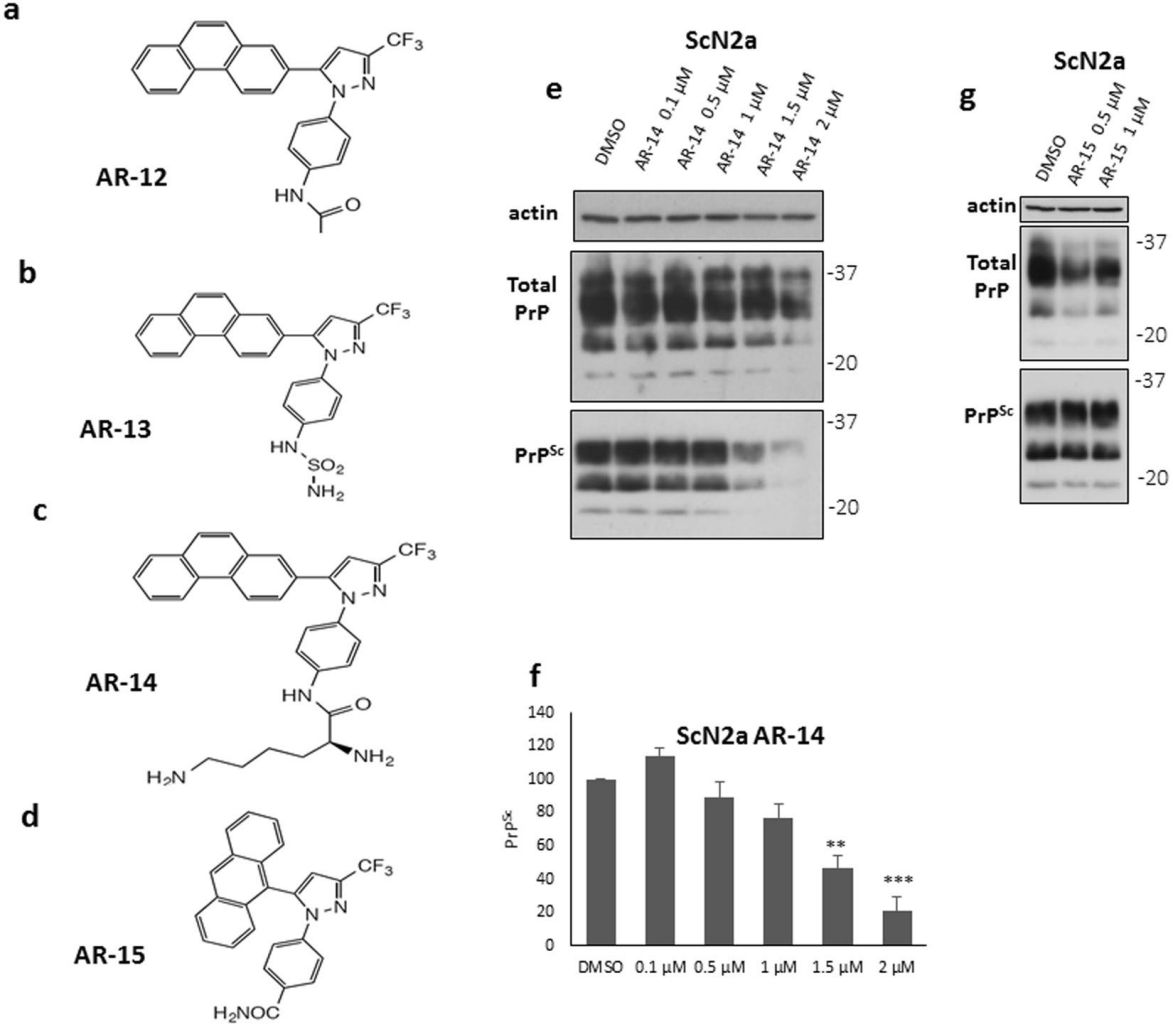
Anti-prion effect of AR-12 analogues. **(a**–**d)** Chemical structures of AR-12, AR-13, AR-14 and AR-15, respectively. **(e)** ScN2a cells were treated for 72 h with various concentrations of AR-14 (0.1, 0.5, 1, 1.5 and 2 µM). Solvent only-treated cells (DMSO) were used as control. Immunoblot was developed with anti-PrP mAb 4H11 and re-probed for actin. **(f)** Densitometric analysis for ScN2a immunoblots. Data are represented as a percentage of vehicle-treated (DMSO) cells. **(g)** ScN2a cells were treated for 72 h with 0.5 and 1 µM of AR-15. Solvent only-treated cells (DMSO) were used as control. Immunoblot was developed with anti-PrP mAb 4H11 and re-probed for actin. (**p < *0*.*01)*, (***p < *0*.*001)* considered significant.

**Figure 3:**
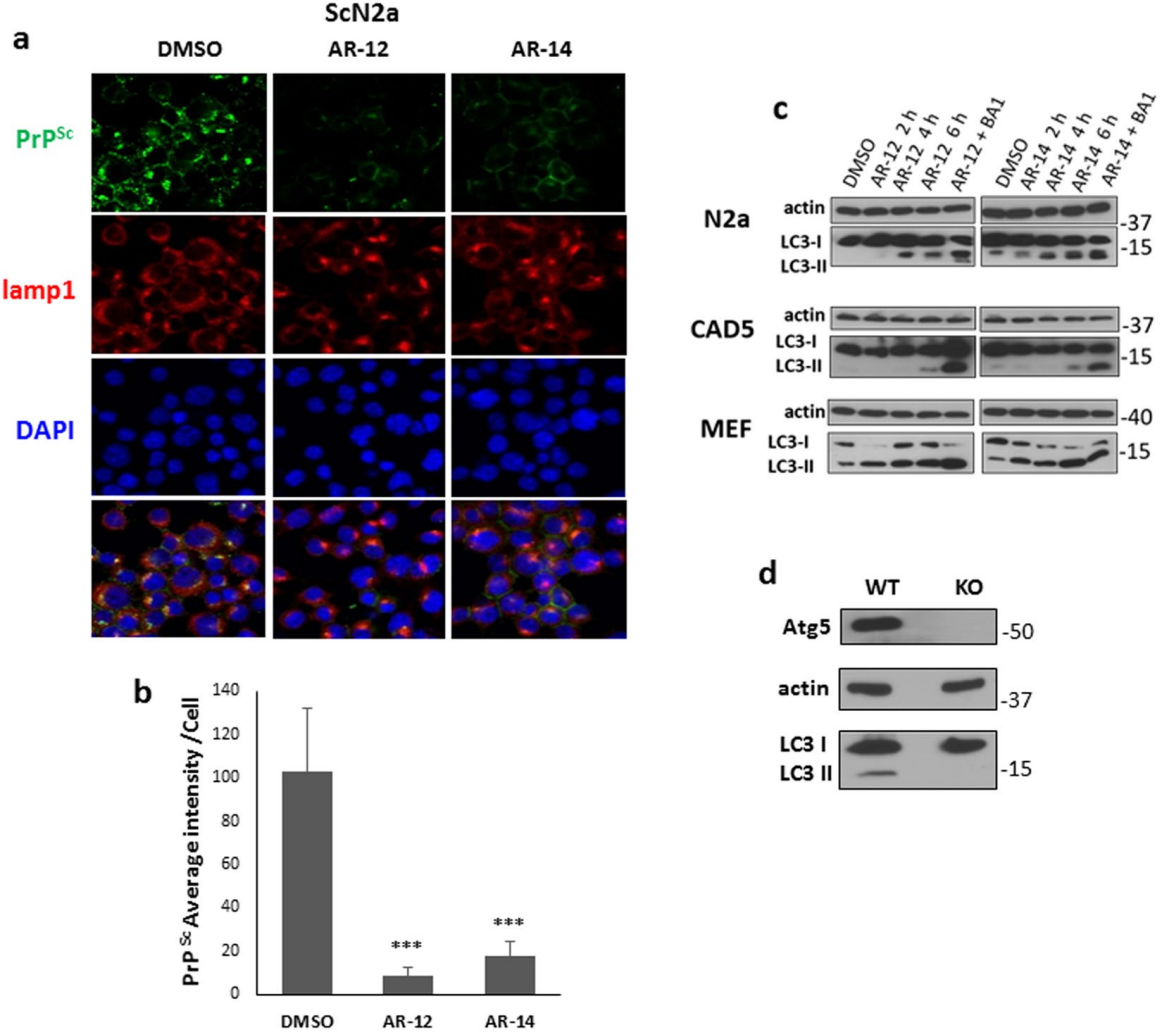
Immunofluorescence analysis of AR-12 and AR-14 effects and induction of autophagy. **(a)** ScN2a cells were treated with AR-12 or AR-14 for 72 h. DMSO-treated cells were used as a control. Cells were fixed and confocal microscopy staining for PrP^Sc^ (mAb 4H11, green) and lamp1 (red) was done. Nuclei were stained with DAPI (blue). Lower panel shows merge. **(b)** The overall immunofluorescence intensity of PrP^Sc^ of five images for either DMSO, AR-12 or AR-14 treated cells was measured. Overall intensity was divided by the cell number contained from the same image quantified by ImageJ “analyze particle” command to calculate the averaged immunofluorescence intensity per cell. **(c)** N2a, CAD5 and MEF cells were treated with AR-12 (3 µM) or AR-14 (2 µM), respectively, for 2, 4 and 6 h, or AR compound plus Bafilomycin A1 (BA1) (100 nm, for 4 h). Solvent only-treated cells (DMSO) were used as control. Immunoblots were developed with anti-LC3 mAb (autophagy marker) and mAb for actin (gel loading control). Treatment both with AR-12 and AR-14 showed a time dependent increase in LC3-II levels. BA1 treated cells had the highest expression level of LC3-II due to the block on autophagic flux and lysosomal function. **(d)** N2a cells were established with knock-out in the autophagy gene ATG5. Immunoblot compares N2a knockout (KO) to wild-type (WT) cells. Immunoblot was probed for Atg5, LC3-I/II and actin. There are no Atg5 and LC3-II bands in N2a-KO cells.

Next, we tested the effect of various available AR-12 analogues on controlling prion infection (AR-13, AR-14, AR-15 and AR-16; Fig. 2b–d). Cytotoxicity assays were conducted first to examine the effect of the test compounds on cell viability. ScN2a and ScMEF cells were treated with different concentrations of each compound for 72 h. For ScN2a cells, AR-14 was non-toxic up to a concentration of 2 µM. LD_50_ was 4 µM **(**Fig. S1d **)**. For ScMEF cells, AR-14 did not show a significant toxicity up to concentration of 3 µM. LD_50_ was 5 µM **(**Fig. S1e **)**. AR-15 was safe to use in a concentration of only 1 µM **(**Fig. S1h **)**. Unfortunately, AR-13 and AR-16 were toxic at the tested concentrations. Consequently, they were excluded from further analysis **(**Fig. S1f,g **)**. To test the effect of AR-14 on PrP^Sc^, ScN2a cells were treated with variable concentrations from 0.1 to 2 µM of AR-14 for 72 h. A significant decrease of PrP^Sc^ was observed after treatment with either 1.5 or 2 µM of AR-14 for 72 h (p < 0.01 and 0.001 respectively). EC_50_ was 1.5 µM **(**Fig. 2e,f **)**. Treatment with 1 µM of AR-15 for 72 h did not show an effect on PrP^Sc^ **(**Fig. 2g **)**. As additional read-out we used PrP^Sc^-specific confocal laser microscopy analysis **(**Fig. 3a,b **)**. In this immunofluorescence analysis, treatment with AR-12 and AR-14 resulted in a significant decrease in PrP^Sc^ compared to vehicle treated cells (p < 0.001). Additionally, a co-staining with the lysosomal marker lamp-1 showed more intense and condensed lysosomes after treatment with AR-12 or AR-14 compared to the diffused staining for lysosomes in cells that received solvent only (DMSO) **(**Fig. 3a **)**.

Collectively, these results indicate that AR-14 has the potential to reduce PrP^Sc^, at even slightly lower concentrations than AR-12.

### Autophagy is involved in the anti-prion effect of AR-12 and AR-14

There is growing evidence that the AR-12 mode of action is related to induction of autophagy^31,42^, thereby, for example, reducing neuritic degeneration caused by amyloid-β peptide^43^. Upon autophagy stimulation, microtubule-associated protein 1 light chain 3 (LC3-I) is post-translationally converted to the lipidated form LC3-II, which is considered as a marker for autophagy. LC3-II is associated with the formation of autophagosomes^44^. To examine the effect of AR-12 and AR-14 on autophagy in our cell culture models, we treated N2a, CAD5 or MEF cells with AR-12 (3 µM) or AR-14 (2 µM) for 2, 4 and 6 hours. An increased level of LC3-II was observed after treatment with AR-12 and AR-14 in all 3 cell lines compared to the vehicle (DMSO) treated cell population **(**Fig. 3c **)**. To exclude that the observed increase in LC3-II upon treatment with AR-12 and AR-14 was a result of impaired autophagic flux, cells were treated with AR-12 or AR-14 plus bafilomycin A1 (BA1). Bafilomycin A1 prevents maturation of autophagic vacuoles by inhibiting fusion between autophagosomes and endosomes/lysosomes and consequently results in accumulation of LC3-II^45^. N2a, CAD5 or MEF cells treated with BA1 together with AR compounds showed higher signals of LC3-II compared to cells treated with AR compounds alone **(**Fig. 3c **)**.

These data show that AR-12 and AR-14 treatment does not block autophagic flux, but stimulates autophagy in these cell lines.

**Figure 4:**
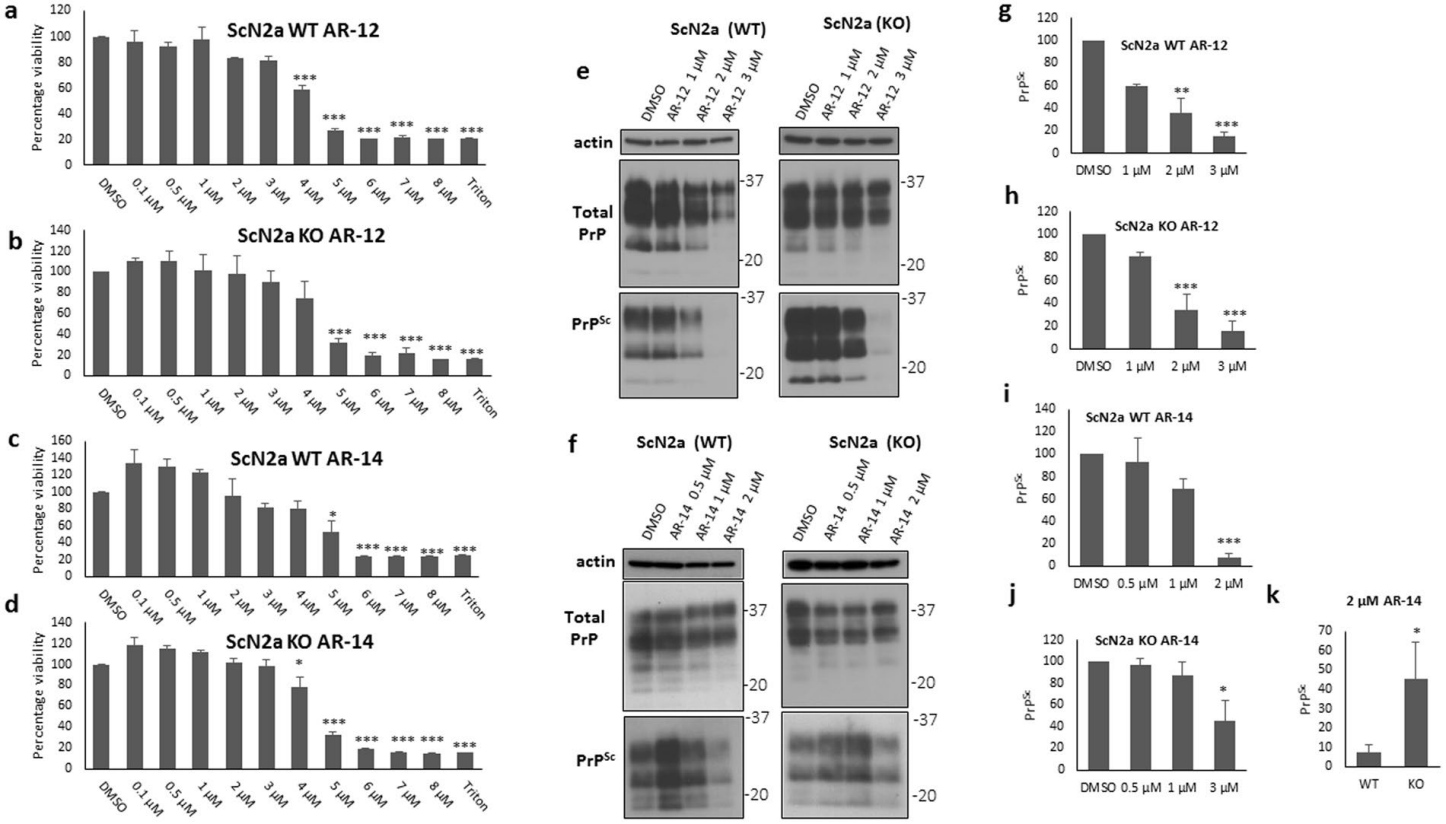
AR-12 and AR-14 anti-prion effects involve autophagy. **(a–d)** XTT viability assay of ScN2a WT and ScN2a Atg5 KO cells treated for 72 h with AR-12 or AR-14. **(e**,**f)** Persistently prion-infected WT (left side) and KO (right side) ScN2a cells (strain 22 L) were treated for 72 h with different concentrations of AR-12 and AR-14, respectively. Solvent only-treated cells (DMSO) were used as control. Immunoblots were developed with anti-PrP mAb 4H11 and re-probed for actin. **(g–k)** Densitometric analysis for panel e and f immunoblots, data were represented as a percentage of vehicle (DMSO) treated cells. (*p < 0.*05*), (***P < 0.*001*) considered significant.

To further shed light on the mechanisms underlying the reduction of PrP^Sc^, we tested the effect of AR compounds on PrP^Sc^ in autophagy-compromised cells. Using CRISPR/Cas9 technology, we had generated Atg5-deficient ScN2a cells (KO) which are defective in autophagy. KO cells do not show any signal for Atg5 or LC3-II in contrast to wild-type (WT) cells **(**Fig. 3d **)**. To test the hypothesis that the intrinsic toxicity of AR-12 and AR-14 was related to autophagy, we performed XTT cytotoxicity assays for WT and KO cells treated with AR-12 and AR-14 for 72 h. We did not observe any remarkable differences in the viability pattern of the treated cells **(**Fig. 4a–d **)**. These data exclude that intrinsic toxicity of AR-12 and AR-14 was related to autophagy. Next, we treated prion-infected WT and KO cells with different concentrations of AR-12 and AR-14 for 72 h **(**Fig. 4e,f **)**. Our immunoblot results showed that AR-12 treatment significantly reduced PrP^Sc^ in both cell populations at concentrations of 2 and 3 µM (P < 0.001). Data were represented as a percentage of control (DMSO) treated cells **(**Fig. 4g,h **)**.

AR-14 treatment reduced PrP^Sc^ significantly in WT and KO cells at 1 and 2 µM concentrations **(**Fig. 4f,i,j **)**. The anti-prion effect of AR-14 was slightly higher in WT cells compared to KO cells **(**Fig. 4f **)**. In wild-type cells, AR-14 (2 µM) was able to significantly decrease PrP^Sc^ to 7.75% (p < 0.001). In KO cells this reduction was only down to 45.7% (p < 0.05), which is a statistically significant difference relative to the 7.75% in WT cells (p < 0.05) **(**Fig. 4i–k **)**. Data were represented as a percentage of control (DMSO) treated cells.

In conclusion, these results indicate an at least partial involvement of autophagy competency in AR-12 and AR-14 mediated anti-prion effects.

### Long-term treatment with AR-12 cured 22L-infected ScN2a cells from prion infection

**Figure 5:**
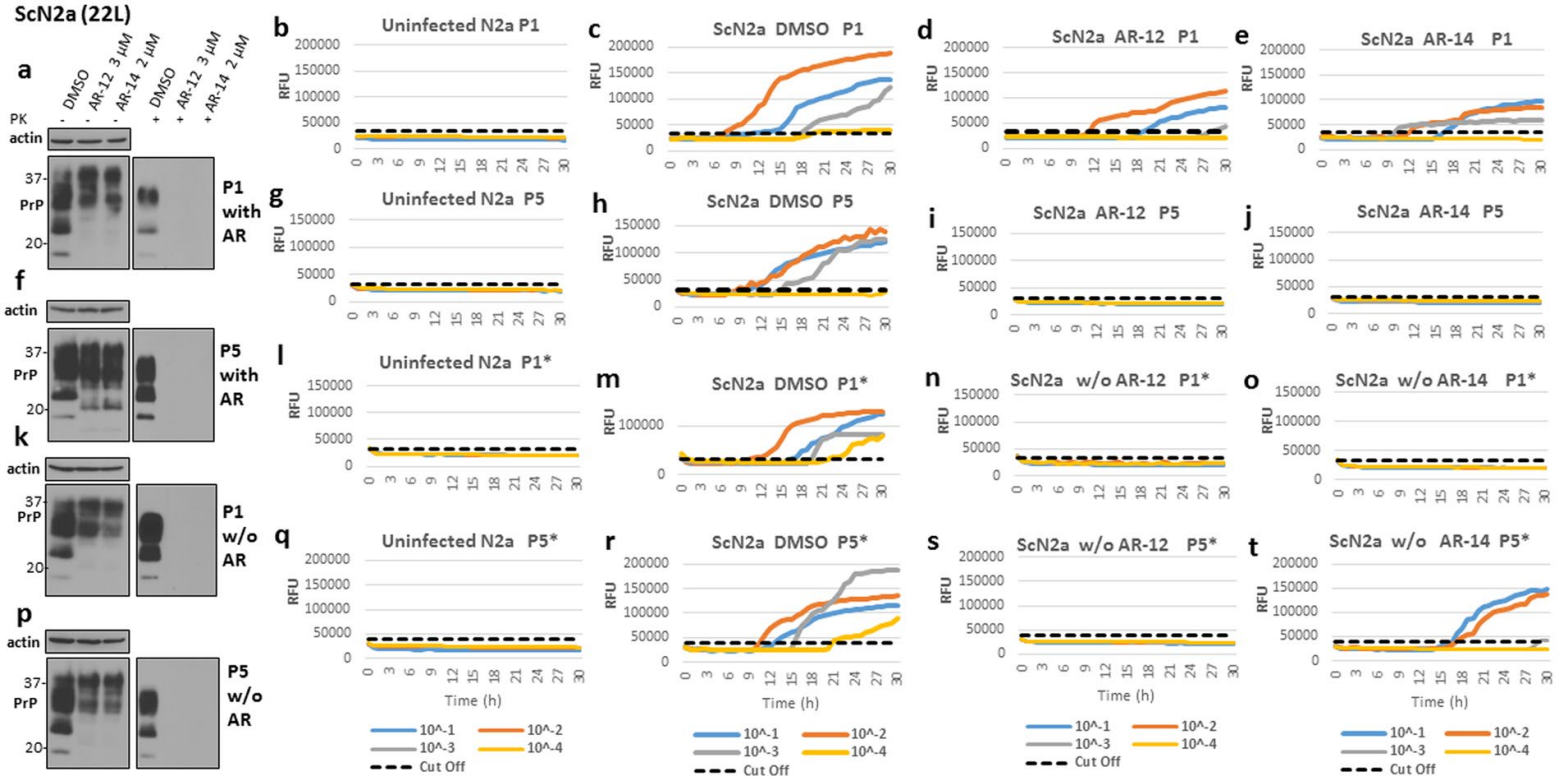
AR-12 long-term treatment cured prion infected ScN2a cells (22 L). ScN2a cells were treated with AR-12 (3 µM) or AR-14 (2 µM). DMSO treatment was used as control. Treatment was continued for five passages (20 days). Then, the treatment was stopped and cells were passaged five times again (20 days). **(a**,**f**,**k** and **p)** Immunoblots showing the effect of treatment with AR compounds on PrP^Sc^ throughout the experiment compared to DMSO treated cells. Immunoblots were developed with anti-PrP mAb 4H11 and probed for actin. **(b**,**g**,**l** and **q)** RT-QuIC analysis for uninfected N2a cells at every passage. Recombinant mouse PrP was used as substrate. Each quadruplicate RT-QuIC reaction was seeded with 2 μl of cell lysate (at dilutions 10^−1^ to 10^−4^). The average increase of Thioflavin-T fluorescence of replicate wells is plotted as a function of time. Y-axis represents relative fluorescent units (RFU) and x-axis time in hours. **(c**,**h**,**m** and **r)** RT-QuIC analysis for DMSO-treated cells. Passages 1 and 5 (P1 and P5) are shown **(c**,**h)**. After discontinuation of the treatment, passages 1 and 5 (P1* and P5*) are shown **(m**,**r)**. **(d**,**i**,**n** and **s)** RT-QuIC-analysis for cells treated with AR-12 (3 µM) for 20 days. Passages 1 and 5 (P1 and P5) are shown **(d**,**i)**. After discontinuation of the treatment, passages 1 and 5 (P1* and P5*) are shown **(n**,**s)**. **(e**,**j**,**o** and **t)** RT-QuIC analysis for cells treated with AR-14 (2 µM) for 20 days. Passages 1 and 5 (P1 and P5) are shown **(e**,**j)**. After discontinuation of the treatment, passages 1 and 5 (P1* and P5*) are shown **(o**,**t)**.

Our short-term treatment with AR compounds revealed a potent reduction of PrP^Sc^ in different prion infected cell lines. Next, we investigated the effect of long-term treatment with AR compounds. We treated ScN2a cells with 3 µM of AR-12 or 2 µM of AR-14 for 20 days, adding the drug with every media change. Cells were passaged five times in the continuous presence of AR-12 or AR-14. Then, treatment was stopped and cells were passaged for another five times, without the test compounds. In addition to immunoblotting as read-out, real-time quaking-induced conversion (RT-QuIC) was used to assess prion seeding activity in AR-treated cells compared to vehicle (DMSO) treated cells. RT-QuIC is a very sensitive *in vitro* prion amplification technique and likely able to detect signs of prion infection in situations where immunoblotting is negative. Uninfected N2a cells were used at every passage as negative control and for calculating the cut-off values **(**Fig. 5b,g,l and q **)**. At passage 1 with AR compounds, immunoblot analysis showed that PrP^Sc^ drastically decreased after treatment with AR-12 or AR-14 **(**Fig. 5a **)**. RT-QuIC analysis showed that in DMSO-treated control cells seeding activity was detected up to a dilution of 10^−3^ **(**Fig. 5c **)**. Cells treated with AR-12 were positive up to a dilution of 10^−2^, paralleled by a strong decrease in seeding activity as evidenced by decrease in relative fluorescence units (RFU) **(**Fig. 5d **)**. Cells treated with AR-14 also were positive up to a dilution of 10^−2^ and showed a decrease in seeding activity for the positive dilutions **(**Fig. 5e **)**. Interestingly, RT-QuIC analysis of postnuclear lysates treated after lysis with AR-12 or AR-14 for 72 h showed less pronounced effects on the inhibition of seeding activity **(**Fig. S2c,d **)** compared to living cells that were treated with AR-12 and AR-14 **(**Fig. 5d,e **)**, confirming that that AR-12 and AR-14 work mainly through an intracellular pathway in living cells. After 20 days (5 passages) of treatment with the AR compounds, PrP^Sc^ was not detectable by immunoblot analysis **(**Fig. 5f **)**. AR-12 and AR-14 treated cells did not show any prion conversion activity in RT-QuIC **(**Fig. 5i,j **)**, whereas DMSO treated cells were strongly positive **(**Fig. 5h **)**. After 20 days of treatment, we passaged cells for another 20 days without any treatment. The first passage showed no prion seeding activity in cells that underwent AR-12 or AR-14 treatment **(**Fig. 5n,o), in contrast to DMSO-treated cells **(**Fig. 5m **)**. Similarly, immunoblot analysis did not show any PrP^Sc^, whereas DMSO-treated cells were clearly positive **(**Fig. 5k **)**. Five passages (20 days) after treatment was discontinued, AR-12-treated cells continued to demonstrate no prion seeding activity **(**Fig. 5s **)**, while DMSO-treated cells did **(**Fig. 5r **)**. However, in AR-14 treated cells prion seeding activity re-appeared, with dilutions 10^−1^ and 10^−2^ positive **(**Fig. 4t **)**. Of note, immunoblot analysis did not show detectable PrP^Sc^ in both AR-12 and AR-14 treated cells compared to DMSO-treated cells **(**Fig. 5p **)**.

Taken together, these data demonstrate that AR-12, but not so much AR-14, is capable of completely clearing prion infection in ScN2a cells infected with 22 L prions.

### Long-term treatment with either AR-12 or AR-14 completely cured ScMEF cells from prion infection

**Figure 6:**
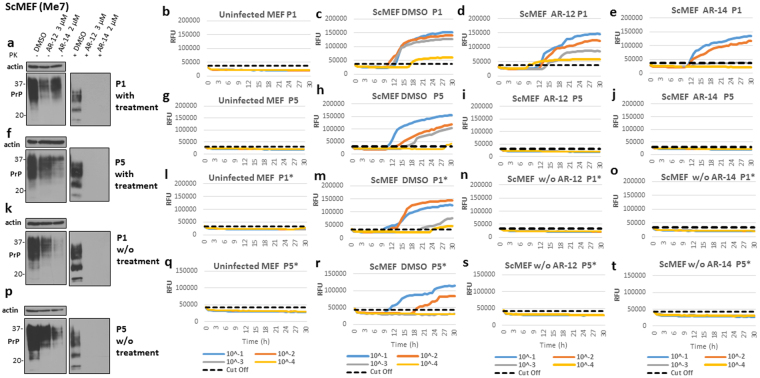
AR-12 and AR-14 long-term treatment cured prion infection in ScMEF cells infected with Me7 prions. **(a–d)** ScMEFs (Me7) were treated with AR-12 (3 µM) or AR-14 (2 µM). DMSO-treated cells were used as a control. Treatment was continued for five passages (20 days). Then, the treatment was stopped and cells were passaged five times again (20 days). **(a**,**f**,**k** and **p)** Immunoblots showing the effect of treatment with AR compounds on PrP^Sc^ throughout the experiment compared to DMSO-treated cells. Immunoblots were developed with anti-PrP mAb 4H11 and re-probed for actin. **(b**,**g**,**l** and **q)** RT-QuIC analysis for uninfected MEF cells at every passage. Each quadruplicate RT-QuIC reaction was seeded with 2 μl of cell lysate (at dilutions 10^−1^ to 10^−4^). The average increase of Thioflavin-T fluorescence of replicate wells is plotted as a function of time. Y-axis represents relative fluorescent units (RFU) and x-axis time in hours. **(c**,**h**,**m** and **r)** RT-QuIC analysis for DMSO-treated cells. Passages 1 and 5 (P1 and P5) are shown **(c**,**h)**. After discontinuation of the treatment, passages 1 and 5 (P1* and P5*) are shown **(m**,**r)**. **(d**,**i**,**n** and **s)** RT-QuIC-analysis for cells treated with AR-12. **(e**,**j**,**o** and **t)** RT-QuIC analysis for cells treated with AR-14.

Subsequently, we investigated the effect of long-term treatment with AR compounds on ScMEF cells. We used the same experimental design as for ScN2a cells **(**Fig. 5 **)**, using immunoblotting and RT-QuIC analysis as read-outs. For RT-QuIC, uninfected cells were used at every passage as negative control and for calculating cut-off values **(**Fig. 6b,g,l and q **)**. At passage 1 with AR compounds, PrP^Sc^ was undetectable after treatment with AR-12 or AR-14 by immunoblot analysis **(**Fig. 6a **)**. Interestingly, in RT-QuIC, these cells continued to show a pronounced prion seeding activity **(**Fig. 6d,e **)**, although their RFUs were lower than the ones of DMSO-treated cells **(**Fig. 6c **)**, with AR-14 being more effective. Twenty days (5 passages) after treatment with AR compounds, PrP^Sc^ was negative in immunoblot analysis **(**Fig. 6f **)**. Additionally, AR-12 and AR-14 treated cells did not show any prion conversion activity in RT-QuIC **(**Fig. 6i,j **)**, while DMSO-treated cells did **(**Fig. 6h **)**. As before, after 20 days of treatment, we passaged cells for another 20 days without any treatment. The first passage showed no prion seeding activity in cells that had received AR-12 or AR-14 treatment **(**Fig. 6n,o **)**, compared to DMSO-treated cells **(**Fig. 6m **)**. In line with this, immunoblot analysis did not reveal any PrP^Sc^, in contrast to DMSO-treated cells **(**Fig. 6k **)**. Five passages (20 days) after the treatment was discontinued, AR-12 and AR-14 treated cells continued to be negative for prion seeding activity **(**Fig. 6s,t **)**, whereas DMSO-treated cells were positive **(**Fig. 6r **)**. Similarly, immunoblot analysis did not show reappearance of PrP^Sc^ in previously AR-12 or AR-14 treated cells **(**Fig. 6p **)**.

Taken together, our data show that both AR-12 and AR-14 were able to clear prion infection in ScMEF cells infected with prion strain Me7.

## Discussion

Prion diseases are fatal infectious neurodegenerative disorders that affect man and animals^6^. In humans, manifestation of disease can be sporadic, genetic or acquired by infection. Human prion diseases are very rare^46^. Nevertheless, sporadic CJD as the main form is found worldwide at a constant incidence, resulting in up to 8,000 fatalities every year. Familial forms are much less frequent, but can be diagnosed long before clinical disease onset, providing a long window for intervention^47^. It will be crucial to understand the molecular requirements for prion propagation, to devise rational strategies for controlling these events, and in the long term, to establish means for treatment to effectively assist individuals incubating these diseases.

An ideal drug candidate should be bioavailable, able to effectively cross the blood-brain-barrier (bbb), have no adverse side effects, and achieve effective therapeutic concentrations in the CNS. As for many other neurodegenerative disorders, such a drug has not been found for prion diseases. Interestingly, a huge variety of compounds with often promising anti-prion activities has been described in *in vitro* systems, with a substantial number still effective in animal models, usually prion-infected rodents, but only four agents were tested in human patients or small-scale human trials^14,15,17,18,48^.

Conceptually, various molecular targets can be selected^17,18^. Most anti-prion compounds interfere in the process of prion conversion, by blocking either PrP^c^ substrate or PrP^Sc^ template. Many strategies target the pool of PrP^c^ amenable to prion conversion, e.g. by shutting-off or decreasing PrP^c^ expression, preventing PrP^c^ cell surface localization, extracting PrP^c^ from the plasma membrane, or by inducing aggregation of PrP^c^ at the cell membrane^17,18^. All these approaches result in a net lack of PrP^c^ eligible for prion propagation. We and others have used the strategy to enhance the intrinsic cellular clearance of PrP^Sc^ ^21,49^. We have reported that the pharmacological induction of autophagy has anti-prion effects *in vitro* and *in vivo* through enhancing the delivery of PrP^Sc^ for lysosomal degradation. Autophagy stimulators such as rapamycin, lithium, trehalose and imatinib have shown anti-prion effects *in vitro* and *in vivo* ^20,21,39,50^. A less used strategy, testable only in animal models, is to block or reverse down-stream effects of prion-induced neurodegeneration, usually summarized under the term neuroprotection. More recently, restoring unfolded protein response has received great attention^15^. It was shown that re-establishing the translational machinery by inhibition of eIF2α-P activity restored memory and prevented features of neurodegeneration. Interestingly, although not entirely new for the prion field, the tested compounds were repurposed drugs, potentially accelerating their investigation in human clinical studies^51^.

The mode of action of the four compounds tested in human subjects is not completely understood^48^. Pentosan polysulfate (PPS), a large polyglycoside molecule, has been shown to inhibit the formation of PrP^Sc^ and had some moderate effects in patients upon intraventricular application^52,53^. Acridine and phenothiazine derivatives like quinacrine and chlorpromazine inhibit prion replication *in vitro* and were tested in human patients, although without clear evidence of efficacy^54–56^. Flupirtine, a nonopioid analgesic with cytoprotective effects and without direct anti-prion activity, showed beneficial effects on cognitive function in some CJD patients^57^. Tetracycline and doxycycline have been shown to interact with PrP^Sc^ and to destabilize the structure of amyloid fibrils^58^, however, effects in humans are questionable^59^.

Given all this, there remains a great need for anti-prion drugs which are effective *in vivo*. AR-12/OSU-03012 is a celecoxib derivative with anti-cancer and anti-microbial activity but unlike celecoxib, AR-12 does not inhibit cyclo-oxygenase (COX)^30,60^, but inhibits several enzymes which may be useful in the treatment of some forms of cancer^61,62^. Additionally, AR-12 has antifungal activity via disruption of phosphoinositide-dependent kinase-1 activity^63^. Previous studies indicated that AR-12 is an autophagy stimulator^31,64^ and recently, it has been shown that AR-12 inhibits amyloid-β peptide-induced neuritic degeneration through autophagy stimulation^43^.

In the present study, we evaluated the effect of AR-12 and its analogues on controlling prion infection in neuronal and non-neuronal cell culture models of prion infection. Short-term application of AR-12 showed a robust decrease of PrP^Sc^ in ScN2a and ScMEF cells infected with different prion strains. Longer application completely eradicated PrP^Sc^ with a complete loss of prion conversion activity in a sensitive RT-QuIC analysis even after discontinuation of the treatment. Short-term treatment with AR-12 significantly decreased PrP^Sc^ in another neuronal cell line, ScCAD5 cells. Synergistic combination therapies might even increase the anti-prion efficacy and circumvent the potential problem of drug resistance, as reported previously^65,66^. Combining AR-12 with phosphodiesterase type 5 (PDE5) inhibitors, such as sildenafil or tadalafil, resulted in enhanced anti-tumor activity^67^. Similarly, combining two or more anti-prion agents may enhance their anti-prion effects and decrease the risk for drug-resistance and drug-induced toxicity. Our future direction is to combine AR-12 with other anti-prion agents and to investigate the effect on PrP^Sc^ and prion seeding activity.

The most likely molecular mechanism underlying the observed anti-prion effect is increased PrP^Sc^ clearance mediated by induction of autophagy. We did not observe any effect of AR-12 treatment on quantity or subcellular distribution of PrP^c^ in non-infected cells **(**Fig. S3 **)**. The very pronounced effect after a 3-day treatment indicates activity on preexisting PrP^Sc^ and cannot be explained with effects on newly formed PrP^Sc^ only. Our data show that AR-12 treatment increased LC3-II in all tested cell lines, in line with induction of autophagy. Additionally, the anti-prion effect of AR-12 was less pronounced in autophagy compromised Atg5-KO cells compared to effects in wild-type cells. Interestingly, ScCAD5 cells showed the lowest induction of the autophagy marker protein LC3-II, which might explain why effects in ScCAD5 cells were weaker. Together, these results demonstrate that induction of autophagy is involved in the anti-prion activity of AR-12.

One of the biggest obstacles in developing treatments for prion diseases is that the compound has to cross the blood-brain barrier, at concentrations that are effective without inducing unwanted side effects. Importantly, AR-12 has been shown to cross the bbb effectively^36^. A phase I clinical trial has been completed and AR-12 has received recently FDA IND-approval for cancer treatment and orphan drug status in Europe for selected indications. This underlines the potential of AR-12 as a drug candidate for counteracting human prion diseases.

AR-14 is a chemical analogue of AR-12. Our study shows that AR-14 could be another potential candidate for drug-induced interference in prion disease. We tested AR-14 in various cell culture models, infected with different prion strains, and we found AR-14 has a robust anti-prion effect in the short-term treatments, even at lower concentrations as AR-12. Of note, treatment with AR-14 increased LC3-II levels and its anti-prion effect was less pronounced in Atg5-KO cells, indicating a role for autophagy in the anti-prion effect of AR-14. Upon extending the treatment with AR-14 in ScMEF cells, AR-14 cleared PrP^Sc^ in immunoblot and prion seeding activity in RT-QuIC analysis. In ScN2a cells, there was reappearance of prion conversion activity at the end of the drug-free period at passage 5. This indicates that although the treated ScN2a cells were negative in both immunoblot and RT-QuIC analysis over a period of almost 40 days, prion infectivity was not completely removed and had the potential to reinitiate prion propagation. Several conclusions can be made from these findings. First, the anti-prion effect of drugs in cell culture is dependent on the chosen cell line model and used prion strain. Second, negativity in immunoblot analysis is easily achieved and does not guarantee absence of PrP^Sc^/prions. This is indicated, for example, by strong signals for RT-QuIC with entirely negative read-outs for immunoblot in passage 1 of AR-treated MEFs in Fig. 6(c–e). RT-QuIC analysis, as expected, represents a much more sensitive read-out than is immunoblot analysis. Third, the duration of treatment is essential, and even a prolonged treatment as done here is no guarantee for clearing cells entirely of prion infection. Unfortunately, most studies perform 5- or 7-day treatments, usually only in one cell model, and use decrease of PrP^Sc^ in immunoblot as parameter for treatment efficacy. Our data clearly show that such an approach can be misleading. Whereas the gold standard, testing for specific prion infectivity of cell lysates in rodent bioassays, is complicated and cannot be done by many laboratories, our study suggests RT-QuIC analysis as viable and easy to perform surrogate methodology.

Due to toxicity concerns in the tested cell lines, AR-13 and AR-16 were excluded in the very early stages from our study. As well, AR-15 could only be used in concentrations that were not sufficient to affect markers of prion infection. Interestingly, it has been published that encapsulation of AR-12 in acetalated dextran (Ace-Dex) microparticles reduced cytotoxicity while retaining the efficiency of the drug, allowing higher drug concentrations for treatment^68,69^. Applying this approach in the future may provide further opportunities for AR-13, 15 and AR-16 in the prion field.

In summary, our study shows that the autophagy stimulator AR-12 and its analogue AR-14 enhance the degradation of PrP^Sc^ and can clear prion seeding activity in cell line models infected with various prion strains. Therefore, they might represent novel therapeutic tools against prion diseases and possibly other protein misfolding diseases. Further work is necessary to establish whether these drugs can provide effective anti-prion effects in rodent models of prion infection, alone or in combination with other drugs.

## Materials and Methods

### Reagents and antibodies

Reagents used were: Proteinase K (PK) (Roche, Germany), Pefabloc inhibitor (Roche, Germany), and Lipofectamine Plus LTX reagent (Invitrogen, USA). Other reagents and chemicals were obtained from Sigma Aldrich, if not otherwise stated. Sources of primary antibodies were as follows: anti-Atg-5 (Clone 7C6; NanoTools, Germany), anti-β-actin (Sigma Aldrich, USA), anti-lamp1 (Cell Signaling, USA) and anti-LC3 (Clone 2G6; NanoTools, Germany). The anti-PrP monoclonal antibody (mAb) 4H11 has been previously described^70^. Peroxidase-conjugated secondary antibodies were from Jackson ImmunoResearch/USA (goat anti-mouse HRP, goat anti-rabbit HRP). AR-12 and its analogues were obtained from ARNO therapeutics.

### Maintenance of cell culture

The mouse neuroblastoma cell line N2a was obtained from ATCC (CCL-131) and was cultured in OptiMEM Glutamax medium (GIBCO, USA) containing 10% fetal bovine serum (Sigma, USA), and penicillin/streptomycin in a 5% CO_2_ atmosphere. CAD5 cells are a central nervous system catecholaminergic cell line^71^. CAD5 cells were subjected to various rounds of single cell sub-cloning and optimized for prion infection^41^. Cells were a generous gift of Dr. S. Mahal (The Scripps Research Institute Florida) and were cultured in OptiMEM Glutamax medium containing 10% bovine growth serum (Hyclone, USA), and penicillin/streptomycin in a 5% CO_2_ atmosphere. Cells were persistently infected with a 1% brain homogenate from terminally-ill mice infected with mouse-adapted scrapie prion strains 22 L or RML. Immortalized mouse embryonic fibroblasts (MEFs) were maintained in DMEM Glutamax medium (GIBCO, USA) containing 10% fetal bovine serum (Sigma, USA).

### Knock-out of *ATG5* in N2a cells by CRISPR/Cas9 technology

Gene-targeting of *ATG5* in N2a cells was done using CRISPR/Cas9 technology as described previously^72^. Briefly, vectors for guide RNA (sgRNA) targeting exons 5 and 6 of the Atg5 gene were purchased from Sigma. The following constructs were used: exon 5: target ID: MM0000476061, vector: U6gRNA-Cas9-2A-GFP, target sequence: CCTCAACCGCATCCTTGGATGG; exon 6, target ID: MM0000476062, vector: U6gRNA-Cas9-2A-RFP, target sequence: GCCATCAACCGGAAACTCATGG. N2a-K21 cells were co-transfected with sgRNA and Cas9 expression vectors by Lipofectamine Plus LTX reagent according to the manufacturer’s protocol. We targeted exons 5 and 6 together in double-transfection. Transient GFP/RFP expression was used as control for transfection efficacy. Single cell clones were obtained and analysed by immunoblotting for Atg5 and LC3-II and by DNA sequencing of individual plasmid clones upon PCR amplification of genomic DNA. Characterization of cells will be described elsewhere (manuscript submitted for publication).

### Proteinase K (PK) digestion and immunoblot

Immunoblot analysis was done as previously described^40^. Briefly, confluent cells were lysed in cold lysis buffer (10 mM Tris-HCl, pH 7.5; 100 mM NaCl; 10 mM EDTA; 0.5% Triton X-100; 0.5% sodium deoxycholate (DOC)) for 10 min. Aliquots of lysates were incubated with PK (20 µg/ml) for 30 min at 37 °C. PK was stopped by addition of proteinase inhibitors (0.5 mM Pefabloc) and directly precipitated with methanol. For samples without PK treatment, proteinase inhibitors were added directly and precipitated with methanol. Precipitated proteins were re-suspended in TNE buffer (50 mM Tris-HCl pH 7.5; 150 mM NaCl; 5 mM EDTA). Samples were run on 12.5% SDS-PAGE, electro-blotted on Amersham Hybond P 0.45 PVDF membranes (Amersham, USA) and analyzed in immunoblot, using Luminata Western Chemiluminescent HRP Substrates (Millipore, USA).

### Cell viability assay

Cells were seeded in 96-well cell culture plates at a density of 1,000 cells per well, then incubated overnight to allow attaching to the plate. The next day, cells were treated with AR-12 and its analogues, or treated with corresponding vehicle solution (DMSO), and incubated for 72 h. Treatment with 1% Triton X-100 was used as a positive control. Cell viability was assessed by TACS® XTT cell proliferation assay (Trevigen, Germany) according to the manufacturer’s instructions. Absorbance was measured at 490 nm using BioTek Synergy HT. Data were expressed as percentage of viability compared with the corresponding control (100%). Concentrations that showed a statistically significant difference from DMSO-treated control cells were considered toxic and consequently were excluded from further analysis.

### Real-time quaking-induced conversion assay (RT-QuIC)

#### Preparation of recombinant protein

Preparation of recombinant prion protein was performed as described^73^. Briefly, mouse PrP (aa 23-231) was cloned into pET-41 plasmids, transformed into *E*. *coli* Rosetta, and bacteria cultured in LB media supplemented with kanamycin (0.05 mg/ml) and chloramphenicol (0.034 mg/ml). The Overnight Express Autoinduction System (Novagen, USA) was used to induce protein expression. Inclusion bodies were isolated from pelleted cells using Bug Buster Master Mix (Novagen, USA) and stored at −20 °C. For purification of recombinant PrP, inclusion bodies were solubilised in guanidine buffer (8 M guanidine-HCl, 100 mM Na-phosphate, 10 mM Tris-HCl, pH 8.0) and incubated on the rocker for 1 h at RT. Ni-NTA Superflow resin beads (Quiagen, USA) were incubated in denaturing buffer (6 M guanidine-HCl, 100 mM Na-phosphate, pH 8.0) for 1 h at RT. Solubilized inclusion bodies were centrifuged at 16,000 × g for 5 min, the supernatant added to the beads and incubated for 1 h with gentle rocking. Beads were then packed into a XK16 glass column (GE Healthcare Life Sciences; USA; length 200 mm). Using an Amersham ÄKTA Explorer FPLC unit running with Unicorn software (5 version, GE Healthcare Life Sciences, USA), protein was refolded by a gradient from 100% denaturing buffer to 100% refolding buffer (100 mM Na-phosphate, 10 mM Tris-HCl, pH 8.0) over 4 h. The column was washed for 30 min with refolding buffer and proteins eluted using a linear gradient from 100% refolding buffer to 100% elution buffer (500 mM imidazole, 100 mM Na-phosphate, 10 mM Tris-HCl, pH 5.8). The central portions of the A280 UV peak were collected into dialysis buffer (10 mM Na-phosphate, pH 5.8). Purified protein was filtered using a 0.22 µm filter, transferred into a Slide-A-Lyzer dialysis cassette (MW 10 kDa; Thermo- Scientific, USA), placed into a 4 liter beaker with dialysis buffer overnight at 4 °C with continuous stirring. Following dialysis, the protein solution was filtered again with a prewashed 0.22 µm Argos syringe filter. Protein concentration was measured using BCA protein assay (Thermo-Scientific, USA), the solution aliquoted and kept in −80 °C until use.

#### RT-QuIC assay

The Real-time Quaking Induced Conversion was performed as described^74^. Briefly, reactions were set up in assay buffer containing 20 mM Na-phosphate, pH7.4, 300 mM NaCl, 1 mM EDTA, 10 μM Thioflavin T and 0.1 mg/ml rPrP substrate. Ninety-eight μl aliquots were added to the wells of a black-walled 96-well optical bottom plate (Nalge Nunc International, Nunc, USA). Tenfold serial dilutions of brain homogenate or cell homogenate were prepared in 0.5 ml microtubes. Quadruplicate reactions were seeded with 2 µl of cell lysate or brain homogenate for a final reaction volume of 100 µl. Reactions contained a final concentration of 0.002% SDS. Plates were sealed with Nunc Amplification Tape (Nalge Nunc International) and incubated in a FLUOstar Omega (BMG Labtech, Cary, NC, USA) plate reader for 30 h. Reactions were incubated at 42 °C, with cycles of 60 s shaking (700 revolutions per minute) and 60 s of rest throughout the incubation. ThT fluorescence measurements (450 nm excitation and 480 nm emission) were taken every 15 min. RT-QuIC data were averaged from four replicate wells and average values plotted against reaction time. Samples were scored positive if at least 50% of replicates reached a ThT fluorescence cut-off, which was calculated based on the average ThT fluorescence plus 5 x standard deviation.

### Confocal microscopy

ScN2a cells at 80–90% confluency were fixed in 4% paraformaldehyde for 30 min at RT, followed by quenching in 50 mM NH_4_Cl, 20 mM glycine at RT for 10 min. Cells were then permeablized in PBS containing 5% FBS and 0.5% Triton X-100 (PBSST) for 30 min before incubation in 6 M GndHCl at RT for 7 min to denature PrP^C^ and generating conditions specific for PrP^Sc^ (epitope retrieval). Cells were incubated with anti-PrP mAb 4H11 at 1:100 and anti-lamp1 at 1:100 (Cell signaling, USA) diluted into PBSST for overnight at 4 °C, Alexa Fluor 488/Cy3 and 594 goat anti-mouse secondary antibody (Jackson Immunoresearch/USA) was used at 1:200 to visualize the immunostaining signal. All quantitative images were captured under the 63x oil lens at the same acquisition settings from a Zeiss LSM 700 confocal microscope. The overall immunofluorescence intensity of PrP^Sc^ from an image was measured from ImageJ software after the background was filtered by the same threshold applied to the same series images. Overall intensity was divided by the cell number obtained from the same image quantified by ImageJ “analyze particle” command to calculate the averaged immunofluorescence intensity per cell.

### Statistical analysis

Statistical analysis was performed using GraphPad Instat (Graph software Inc., V 3.05, Ralf Stahlman, Purdue Univ.) using either the unpaired two-tailed *t-test* for pair-wise comparisons or the *One-way ANOVA* analysis with *Tukey post test* for multiple comparisons. Statistical significance for all the experiments was expressed as (Mean ± SEM). *p < 0.05, **p < 0.01, ***p < 0.001 considered significant. All the western blot figure were cropped using PowerPoint 2013 program.

## Electronic supplementary material

Supplementary Figures
