# Supplementary material for: The celecoxib derivatives AR-12 and AR-14 induce autophagy and clear prion-infected cells from prions
Source: Sci Rep. 2017 Dec 14;7:17565. doi: 10.1038/s41598-017-17770-8 (PMC5730578; doi:10.1038/s41598-017-17770-8)
Supplement: Supplementary file 1 — Supplementary Figures [file 41598_2017_17770_MOESM1_ESM.pdf]

The celecoxib derivatives AR-12 and AR-14 induce autophagy and clear prion-infected cells from prions.

Basant A. Abdulrahman, Dalia H. Abdelaziz, Simrika Thapa, Li Lu, Shubha Jain, Sabine Gilch, Stefan Proniuk, Alexander Zukiwski, and Hermann M. Schatzl

Supplementary Data

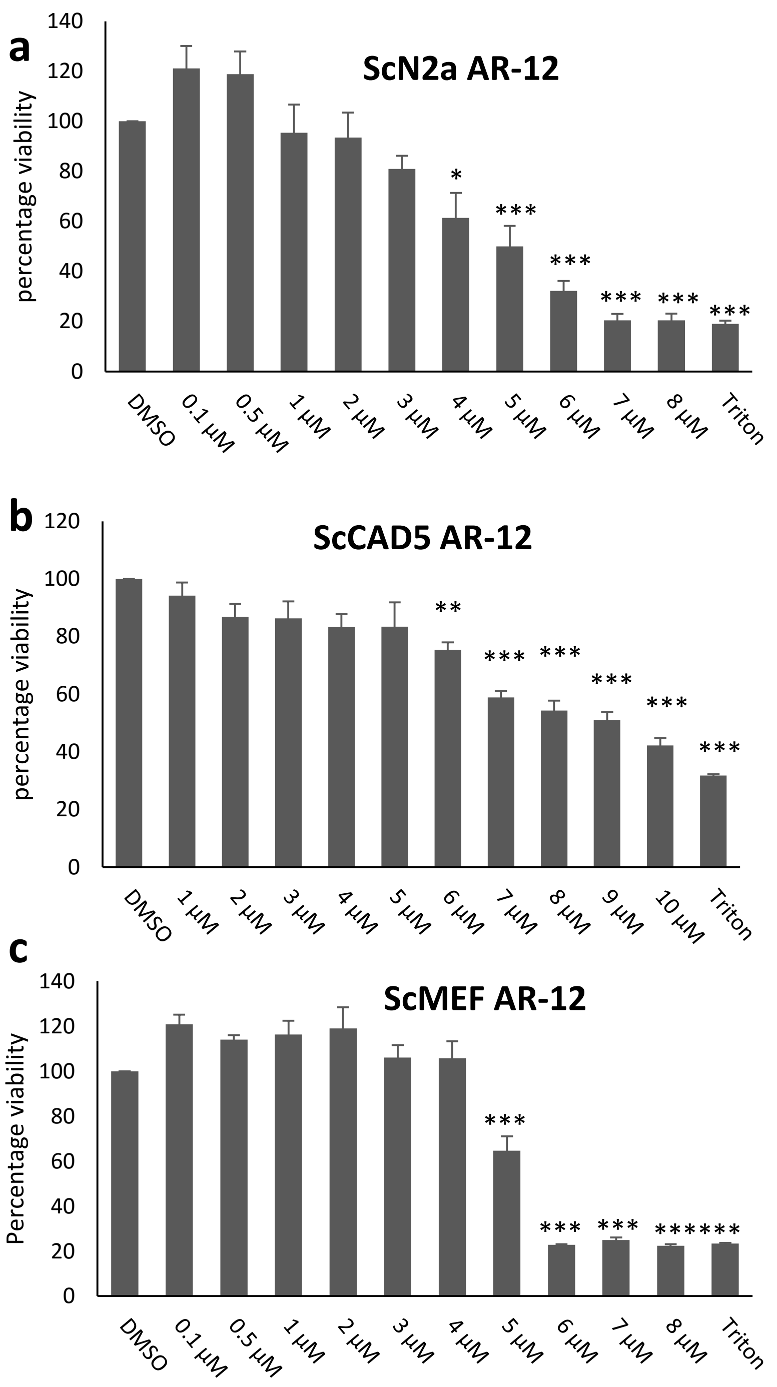

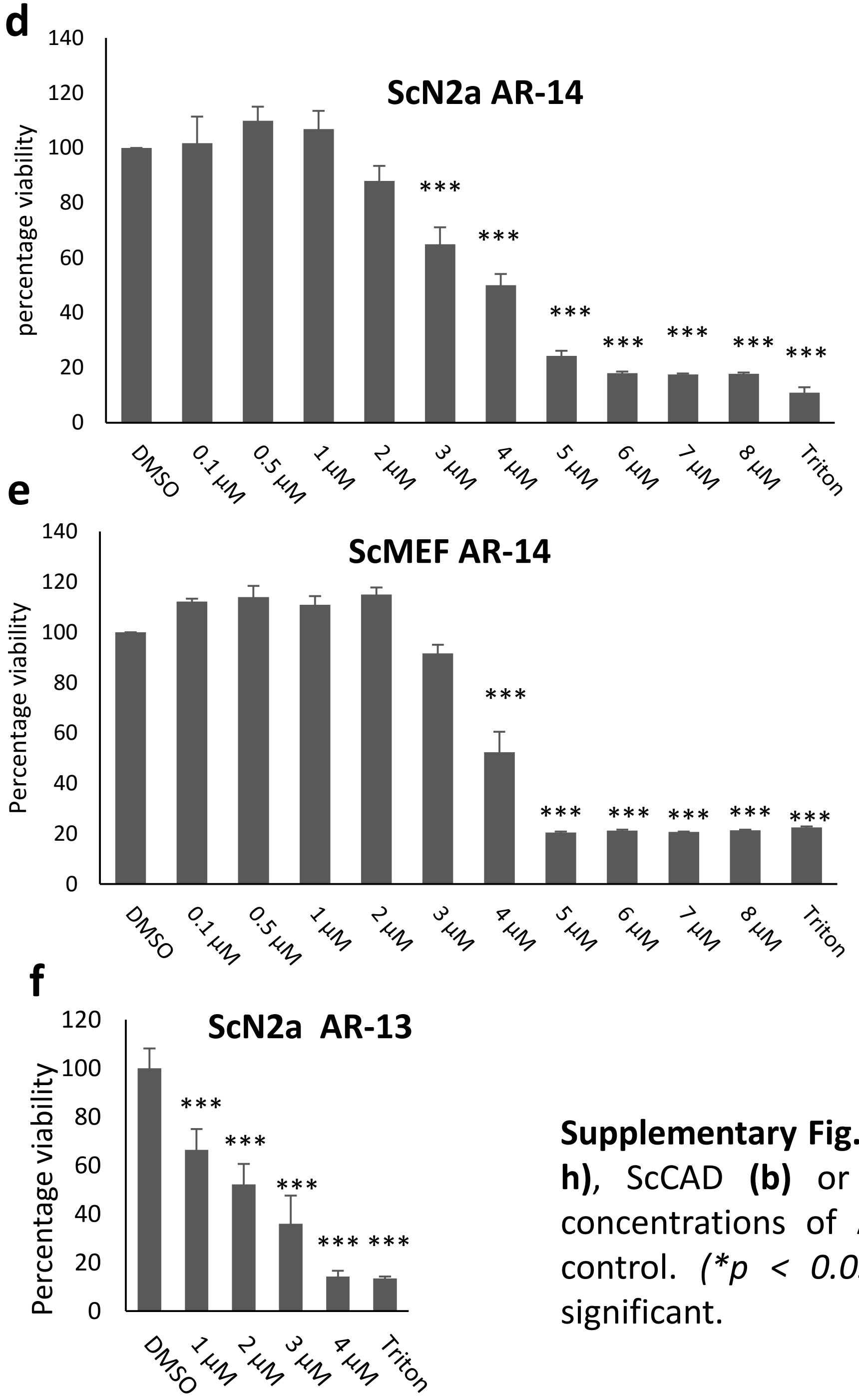

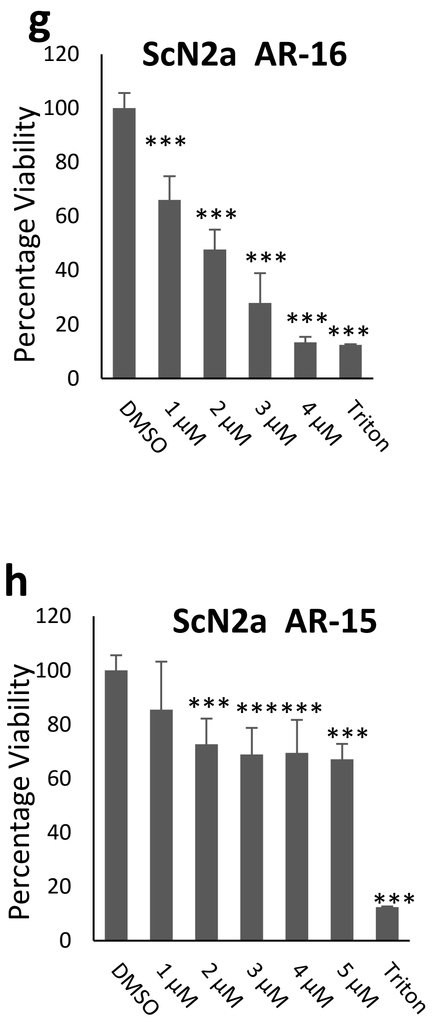

Supplementary Fig. 1: XTT viability assay for ScN2a **(a, d, f, g and h)**, ScCAD **(b)** or ScMEF cells **(c, e)** treated with different concentrations of AR compounds. DMSO was used as vehicle control. *(*p < 0.05), (** p<0.01), (*** p< 0.001)* considered significant.

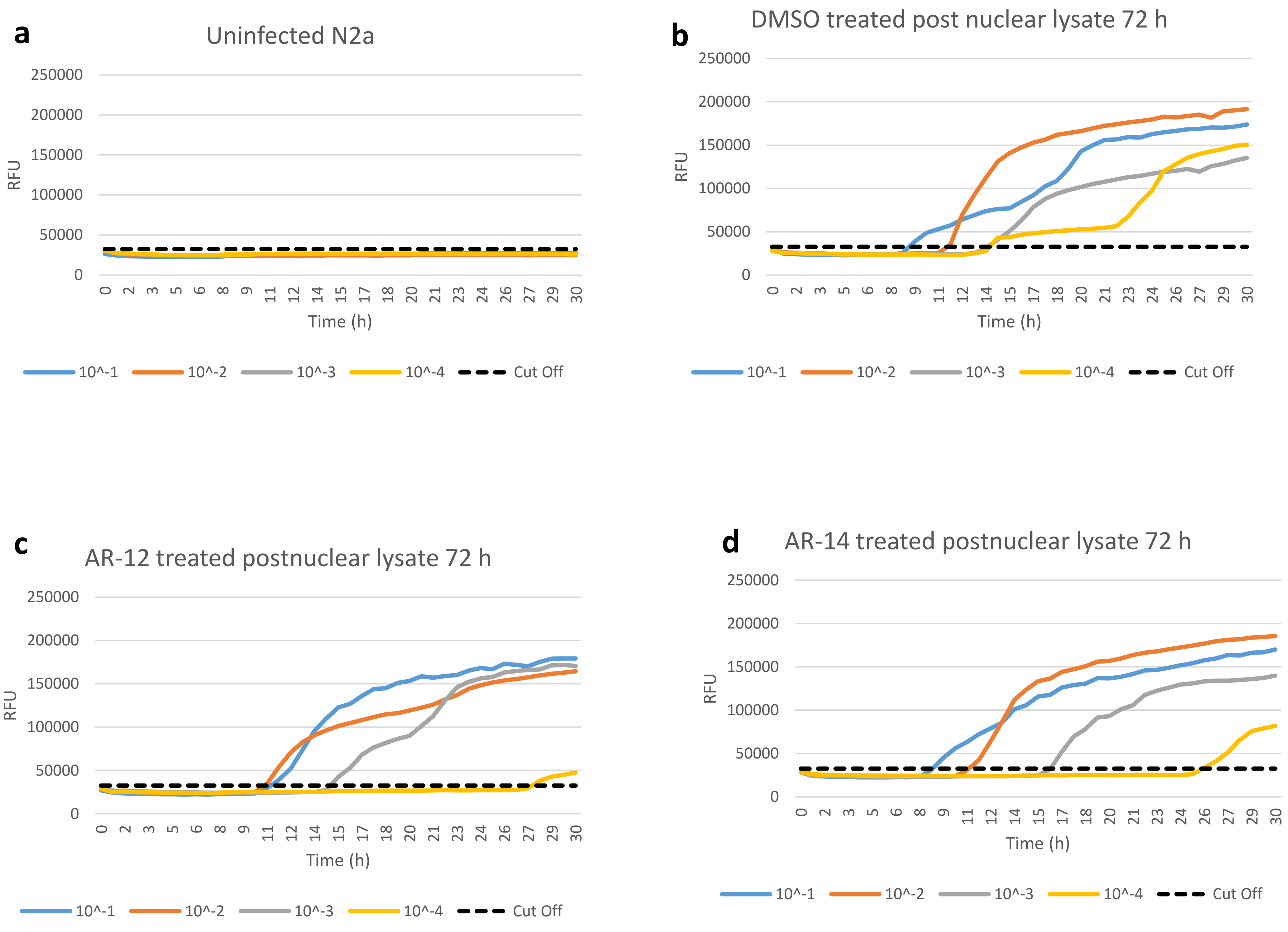

Supplementary Fig. 2:
RT-QuIC analysis of post-nuclear lysates of either uninfected N2a cells **(a)** or post-nuclear lysates of ScN2a cells treated after lysis with AR-12 (3 µM) **(c)**, AR-14 (2 µ) **(d)** or DMSO **(b)** for 72 h. Each quadruplicate RT-QuIC reaction was seeded with 2 µl of post-nuclear lysate (at dilutions 10^{-1} to 10^{-4}). The average increase of Thioflavin-T fluorescence of replicate wells is plotted as a function of time. Y-axis represents relative fluorescent units (RFU) and x-axis time in hours (h).

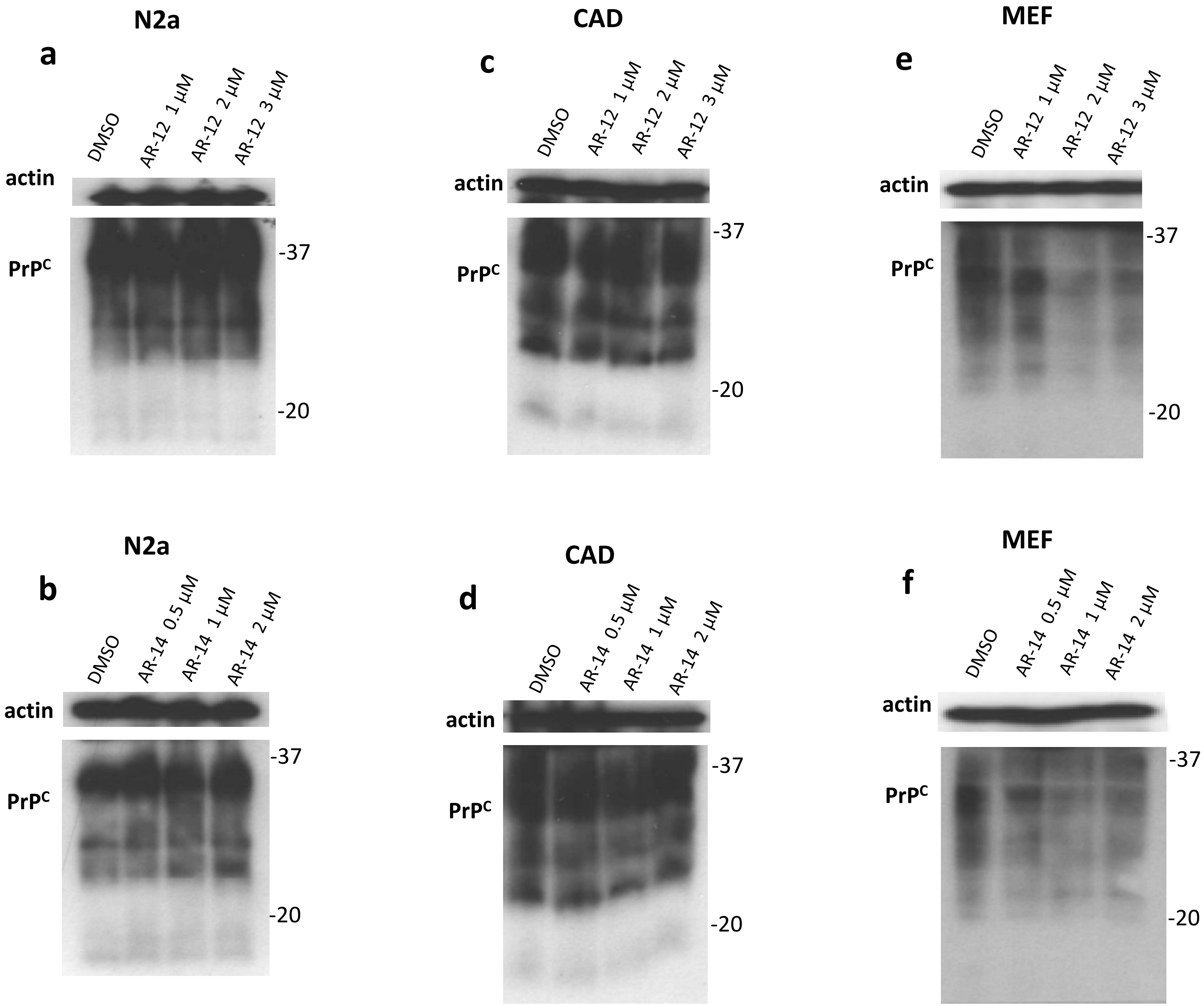

Supplementary Figure 3: Uninfected N2a **(a, b)**, CAD **(c, d)** and MEF **(e, f)** cells were treated with AR-12, AR-14 or DMSO as a control for 72 h. Immunoblots were developed with anti-PrP mAb 4H11 and re-probed for actin.
